# High Representation of Archaea Across All Depths in Oxic and Low-pH Sediment Layers Underlying an Acidic Stream

**DOI:** 10.3389/fmicb.2020.576520

**Published:** 2020-11-17

**Authors:** Marco A. Distaso, Rafael Bargiela, Francesca L. Brailsford, Gwion B. Williams, Samuel Wright, Evgenii A. Lunev, Stepan V. Toshchakov, Michail M. Yakimov, David L. Jones, Peter N. Golyshin, Olga V. Golyshina

**Affiliations:** ^1^School of Natural Sciences, Bangor University, Bangor, United Kingdom; ^2^Centre for Environmental Biotechnology, Bangor University, Bangor, United Kingdom; ^3^School of Agriculture and Environment, The University of Western Australia, Perth, WA, Australia; ^4^Institute of Living Systems, Immanuel Kant Baltic Federal University, Kaliningrad, Russia; ^5^National Research Centre “Kurchatov Institute”, Moscow, Russia; ^6^Institute for Biological Resources and Marine Biotechnology, CNR, Messina, Italy

**Keywords:** acidophilic archaea and bacteria, Thermoplasmatales, “*Candidatus* Micrarchaeota”, unclassified Euryarchaeota/Terrestrial Miscellaneous Euryarchaeotal Group, acid mine drainage systems, mine-impacted environments, sediment microbiome

## Abstract

Parys Mountain or Mynydd Parys (Isle of Anglesey, United Kingdom) is a mine-impacted environment, which accommodates a variety of acidophilic organisms. Our previous research of water and sediments from one of the surface acidic streams showed a high proportion of archaea in the total microbial community. To understand the spatial distribution of archaea, we sampled cores (0–20 cm) of sediment and conducted chemical analyses and taxonomic profiling of microbiomes using 16S rRNA gene amplicon sequencing in different core layers. The taxonomic affiliation of sequencing reads indicated that archaea represented between 6.2 and 54% of the microbial community at all sediment depths. Majority of archaea were associated with the order Thermoplasmatales, with the most abundant group of sequences being clustered closely with the phylotype B_DKE, followed by “E-plasma,” “A-plasma,” other yet uncultured Thermoplasmatales with *Ferroplasma* and *Cuniculiplasma* spp. represented in minor proportions. Thermoplasmatales were found at all depths and in the whole range of chemical conditions with their abundance correlating with sediment Fe, As, Cr, and Mn contents. The bacterial microbiome component was largely composed in all layers of sediment by members of the phyla Proteobacteria, Actinobacteria, Nitrospirae, Firmicutes, uncultured Chloroflexi (AD3 group), and Acidobacteria. This study has revealed a high abundance of Thermoplasmatales in acid mine drainage-affected sediment layers and pointed at these organisms being the main contributors to carbon, and probably to iron and sulfur cycles in this ecosystem.

## Introduction

Parys Mountain (Parys Mt) or Mynydd Parys (Isle of Anglesey, United Kingdom) is an abandoned copper mine which contains abundant sulfidic deposits in the form of pyrite, chalcopyrite, sphalerite, and galena minerals. As with many other low pH environments associated with metal mining activity, the site is characterized by the presence of acidic streams or acid mine drainage (AMD) waters, which result from the oxidative dissolution of sulfidic minerals (Johnson, 2012). Like other AMD systems, Parys Mt streams contain large concentrations of dissolved metals and metalloids which constantly flow into the Irish Sea resulting in marine pollution (Johnson, 2012). This site attracts continuous scientific interest, as reflected in the large number of studies and the identification of many new species of acidophilic bacteria and archaea (Johnson et al., 2014; Jones and Johnson, 2015; Golyshina et al., 2016b).

Our earlier study on microbial assemblages in AMD water and sediments taken from the surface of one of acidic streams of Parys Mt revealed that archaea dominated the microbial community (Korzhenkov et al., 2019). Archaea affiliated with Euryarchaeota constituted the major group (67%) of the total shotgun reads in the community. One particular group of sequences associated with still uncultured archaea of the order Thermoplasmatales (similar to “E-plasma” metagenomic variant) was shown to represent 58% of all metagenomic reads. In the upper sediment layer, bacterial representatives (33%) were mostly related with Proteobacteria. Other bacterial reads present in low amounts (2–6%) were largely affiliated with Actinobacteria, Nitrospirae, Bacteroidetes, Acidobacteria, and Firmicutes (Korzhenkov et al., 2019).

However, in the lotic community, Proteobacteria, Nitrospirae, Acidobacteria, and Actinobacteria did collectively outnumber archaea (Korzhenkov et al., 2019).

The populations of microorganisms inhabiting sediments in AMD-affected areas have been the subject of numerous studies (Kock and Schippers, 2008; Sánchez-Andrea et al., 2011, 2012; Sun et al., 2015; Zhang et al., 2019 and others). These works established that bacteria were highly abundant in AMD sediments and thus assumed they were mainly responsible for biogeochemical cycling in these ecosystems. For example, only low numbers of archaea were reported in sediments of mine tailing dumps in Botswana, Germany, and Sweden and only in oxidized zones (Kock and Schippers, 2008). Although archaea of the order Thermoplasmatales are well-known inhabitants of AMD environments, including sediments, these organisms were found to be present in very low abundance and thus assumed to be unimportant (Kock and Schippers, 2008; Sánchez-Andrea et al., 2011, 2012; Sun et al., 2015; Zhang et al., 2019). Frequently however, the detailed information about the archaeal component is missing, or archaea were excluded from the analysis, leading to a potential underestimation of the ecological role of archaea in AMD ecosystems (Wakelin et al., 2012; Lukhele et al., 2019). To understand the patterns of archaeal distribution in sediments of an acidic stream at Parys Mt and to assess their potential role in elemental cycling, we collected shallow sediment cores (0–20 cm depth) from the AMD stream. We used a combination of chemical analysis and SSU rRNA gene amplicon sequencing to resolve, layer-by-layer, microbial composition changes with depth and across the chemical gradient in order to understand whether particular geochemical factors were associated with archaeal abundance and to assess their functional role *in situ*.

## Materials and Methods

Sampling was conducted in the acidic stream located at Parys Mt (GPS location 53.38708°−4.34968°) as described previously (Supplementary Figure 1; Golyshina et al., 2016a,b; Korzhenkov et al., 2019). Intact sediment cores were taken in September 2018 at three random locations each near another (within 15 cm distance) using polycarbonate tubes (50 cm-long with inner diameter of 4 cm). The tubes were gently pressed by hand into the sediment, then plugged with a butyl rubber stopper at the top. The intact cores were then carefully removed and the base of the tubes plugged with another butyl stopper and subsequently transported back to the laboratory for analysis. Upon arrival (ca. 40 min after sampling), the cores were sliced into 2–3 cm-thick disks and transferred into sterile polypropylene 50 ml Falcon tubes for consequent chemical and microbiological analyses. pH and Eh potential in the sediment surface layers were measured in the field using a SevenGo multimeter (Mettler-Toledo, Leicester, United Kingdom) and then again in the cores on return to the laboratory.

### DNA Extraction and 16S rRNA Gene Amplicon Sequencing

DNA was extracted from 0.25 g of soil sample from each layer of three cores using the DNeasy PowerLyzer PowerSoil kit (QIAGEN) according to manufacturer’s instructions. Two independent DNA extractions were carried out for each sample. Quality and concentration of extracted DNA were assessed by gel electrophoresis and by Qubit 4.0 Fluorometer dsDNA BR Assay Kit (Life Technologies, United States).

Libraries of 16S rRNA gene amplicons were prepared by single PCR with double-indexed fusion primers as described previously (Fadrosh et al., 2014). Hypervariable V4 16S rRNA gene fragment was amplified using modified forward primer F515 (5′-GTGBCAGCMGCCGCGGTAA-3′) and reverse R806 prokaryotic primer (5′-GGACTACHVGGGTWTCTAAT-3′), which amplify an approximately 290 bp region. Primers were designed to contain: the Illumina adapters and sequencing primers, a 12 bp barcode sequence, a heterogeneity spacer to mitigate the low sequence diversity amplicon issue, and 16S rRNA gene universal primers (Fadrosh et al., 2014). PCRs were performed using MyTaq Red DNA Polymerase (Bioline). All reactions were run with no-template negative controls. Thermocycling conditions were: initial denaturation at 95°C for 2 min, followed by 30 cycles at 95°C for 45 s, 50°C for 60 s, and 72°C for 30 s with a final elongation at 72°C for 5 min. Amplicons were visualized in a 1.5% tris-acetate agarose gels using a GelDoc System (Bio-Rad, CA, United States). DNA bands of approximately 440 bp were gel-purified using QIAEX II Gel Extraction Kit (QIAGEN).

The purified amplicons were then quantified using Qubit 4.0 Fluorometer (Life Technologies, Carlsbad, CA, United States), pooled in equimolar amounts and the final pool was run on Illumina MiSeq platform (Illumina, San Diego, CA, United States) using 500-cycle v2 chemistry (2 × 250 bp paired-end reads) at the Centre for Environmental Biotechnology, Bangor, United Kingdom.

### Bioinformatic Analysis

Raw sequencing reads were processed according to previously described protocols (Fadrosh et al., 2014; Korzhenkov et al., 2019). Briefly, the data was pre-processed in order to extract the barcodes from sequences, and then cleaned of primer sequences using tagcleaner. The barcodes and the sequences were re-matched again using in-house Python scripts. The resulting filtered reads were analyzed using QIIME v1.3.1. First, the libraries were demultiplexed based on the different barcodes. Then, the sequences were classified on operational taxonomic units (OTUs) combining *de novo* and reference-based methods (open-reference OTU generation algorithm) using the SILVA version 132 reference database.

In the case of OTUs assigned to order Thermoplasmatales, a further taxonomic assignation analysis was performed using a local Blast (Camacho et al., 2008) database based on a selection of 42 reference sequences, running a final individual blast against *nr* database for those OTU sequences with <97% of identity in their best hit against the local database.

### Statistical Analysis

All statistical analysis and figures were generated using the R programming language (R Development Core Team, 2008). Principal components analysis (PCA) was undertaken using the *prcomp* function form package *stats*, included on basic R core. In the case of the non-metric multidimensional scaling (NMDS) analysis, we used the *vegan* package (Oksanen et al., 2019). For canonical correlation analysis (CCorA) internal R scripts were developed, using basic R functions.

### Phylogenetic Analysis of Archaea

For phylogenetic tree construction, we selected those OTU sequences assigned to Archaea with more than 100 reads along the three cores and also 34 reference sequences belonging to different groups. Multiple alignment of sequences was developed using *Mafft* (Katoh and Standley, 2013). *UGENE* (v 1.9.8) was used for the trimming of the extremes and trimAL (Capella-Gutierrez et al., 2009) for internal trimming of the alignment, removing columns with gaps on more than the 20% of the sequences or with similarity scores lower than 0.001, producing a final multiple alignment of 293 positions. Phylogenetic tree was calculated by maximum likelihood with bootstrapping of 1,000 replicates.

## Chemical Analysis

### Background Chemical Analysis

Cores were divided by layers and subsamples removed for physicochemical analysis. Moisture content was determined for the <2 mm fraction by drying at 105°C for 24 h. The organic matter content of the sediment was measured using the loss-on-ignition method, in a muffle furnace (450°C, 16 h; Ball, 1964). Sediment C and N content was determined after oven-drying (105°C, 24 h) using a TruSpec CN analyzer (Leco Corp., St Joseph, MI, United States). Bulk elemental analysis on the dried, sieved fraction (40°C, <125 μm) was undertaken by total reflection X-ray fluorescence (TXRF) using a Bruker S2 Picofox TXRF spectrometer (Bruker Inc., MA, United States). Ion chromatography (IC) was used to determine anion concentrations (F^–^, Cl^–^, NO_3_^–^, PO_4_^3–^) in 1:10 (w/v) sediment: E-pure water (18 MΩ resistance) extracts using a 930 Compact IC Flex (Metrohm, Herisau, Switzerland).

### Analysis of Black Layers (Oily Deposits) Within the Sediment

Two samples of sediment layers with an oily appearance and hydrocarbon-like odor were selected for further analysis. Samples were weighed out in aliquots of around 100 mg for extraction. The method of extraction was modified from the EPA 3550C method for extraction of non-volatile and semi-volatile organic compounds from solids such as soils, sludges, and wastes by ultrasonic extraction (USEPA, 2007). Briefly, an equal amount of anhydrous sodium sulfate was mixed with the sample to form a free-flowing powder. The sample was then spiked with an internal standard (10 mg pristine) and extracted using 0.5 ml of a 1:1 (v/v) acetone:chloroform solution. The extraction was assisted by the use of an ultrasonic bath. The sample tube was suspended in the bath at room temperature for 1 min. After extraction, the sample was separated by centrifugation, the supernatant retained, and the pellet extracted a second time as described above. The combined organic fractions were merged and evaporated to dryness at room temperature with a gentle stream of nitrogen. Once dry, the sample was resuspended in 200 μl of ethyl acetate and filtered (0.22 μm) prior to analysis.

Analysis was undertaken using a Perkin Elmer Clarus 500/580 GC-MS with a HP-5ms column (30 m, 250 μm ID and 25 μm film thickness). The carrier gas was helium, the split ratio set at 10:1, while the temperatures for the inlet, transfer line, and ionization source were 250, 180, and 200°C, respectively. The detector was set to scan between 80 and 500 μ with a 3 min solvent delay. The initial oven temperature was 60°C (10 min) followed by an 8°C/min ramp to 300°C followed by a 10 min hold. Approximate quantification of the analytes was achieved by comparing peak area to that of pristane and a response factor of 1 assumed. For pristine, a 6-point calibration curve was made between 0.5 and 50 μg/ml. Retention times of the unbranched alkanes were determined using a standard mixture of C_10_–C_19_.

## Results and Discussion

### Physicochemical Data

Cores 1, 2 and 3 showed slightly different values in pH and redox potential. Cores 1 and 2 showed a similar tendency in increasing pH with depth from 1.65–1.7 (surface) to 2.4 at a depth of 8 cm in Core 1 and to 2.68 at a depth of 15 cm for Core 2. Redox was found to be positive in all layers with insignificant variations between depths and with values always >400 mV (range 413–470 mV). The three cores had visual differences in structure and exhibited mostly “oxidized colors,” from mixtures of yellow/brown, to red/brown with some ochre and in some places a completely black appearance. Core 3 was distinct in comparison to other cores, being more homogeneous and with a stable pH (2.4–2.5) across the whole depth gradient (Supplementary Table 1).

Comparison of physical–chemical parameters between cores suggested certain variations in the content of metals and metalloids, anions, nitrogen, and organic matter (Supplementary Table 1). Core 1 possessed more Fe and Pb in the three upper layers (1.1, 1.2, 1.3.1) and a consistently high presence of As in all layers. Core 2 demonstrated more Rb and Ti in all layers. Both Cores 1 and 2 showed an increase in Al with depth. In contrast, Core 3 exhibited high concentrations of Cu in two layers (3.4 and 3.6, depth 9–11 and 19–21 cm), Zn (layers 3.5 and 3.6, depth 13–16 and 19–21 cm) and Rb (layers 3.3 and 3.4, depth 6–9 and 9–11 cm).

The highest amounts of organic matter were measured for Core 1 (layers 1.2 and 1.3.1) and Core 3 (layers 3.1, 3.5, and 3.6). Core 2 was found to have a low organic matter content in the sediment. The total amount of N was found to be higher in Core 2 (layers 2.2, 2.3, and 2.4) and in Core 3 (3.3, 3.4, 3.5, and 3.6). The C:N ratio was significantly higher in upper layers of Core 1 (values of 25.4 and 12.5 for layers 1.1 and 1.2, respectively) and Core 2 (26.7). In Core 3, an opposite pattern was apparent with C:N ratios of 12.4 and 17.6 seen in the deeper layers (3.6 and 3.7).

Interestingly, few fluctuations were observed in the content of fluoride, chloride, nitrate, phosphate, and sulfate. Core 1 (layer 1.3.2) possessed the highest concentrations (in mg/kg) of F^–^ (65.3), Cl^–^ (6.5), NO_3_^–^ (653), and SO_4_^2–^ (90528). Core 3 exhibited an increased content of F^–^, PO_4_^3–^, and SO_4_^2–^ in some layers (Supplementary Table 1). These observations suggest a high degree of heterogeneity in chemical composition between the cores and individual subsamples.

**FIGURE 1:**
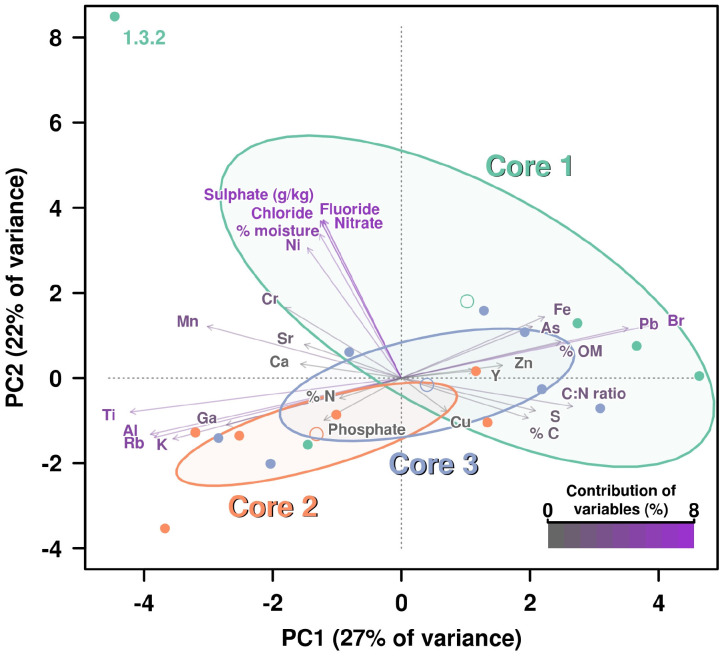
PCA including all chemical parameters analysis by principal components analysis (PCA) of the influence of all chemical properties measured on the three cores. Contribution of each variable (chemical properties) to this graphical representation is shown by a color key from medium gray (less contribution) to violet (highest contribution). Ellipses and open dots represent the variance and mean for each core, respectively. Anion concentrations are showing the highest percentages of contribution due to the higher figures on these values for measured on layer 1.3.2, which is disrupting the variance (ellipse) corresponding to Core 1.

We analyzed 31 different chemical properties in the sediments which we divided into three categories, namely: “Carbon-Nitrogen,” “Anions,” and “Other elements.” A preliminary PCA showed a very complex distribution of the influence of chemical variables over the different core layers. Also, some of the chemical variables overlapped and were not used in order to reduce redundancy. Measures of total C and N (mg/kg) were removed from the analysis, while sulfate (g/kg) was included. Therefore, 28 of 31 chemical properties were included in the analysis. The analysis was divided into three different parts according to each type of chemical property. Each of these analyses is composed of a PCA where the contribution percentage of each variable has been calculated and included using a color key, specific for each core (Figure 1 and Supplementary Figures 2–4).

The PCA for “Carbon-Nitrogen” showed that Core 1 and 2 are quite distinct from each other while Core 3 remained in an intermediate position (Supplementary Figure 2).

Principal components analysis demonstrated that variance for F^–^, Cl^–^, NO_3_^–^ and SO_4_^2–^ are much higher on the sample 1.3.2 than for the rest of the layers, being so different those four measures overlapped on the representation. The concentration of PO_4_^3–^ was the variable that contributed the most to the distribution of the samples in the PCA. Concentration of PO_4_^3–^ was below the limit of detection (0.1 mg/kg) in every layer of Core 1, was detected in 2 layers out of 6 of the Core 2 in concentration <1 mg/kg (dry sediment), whereas 4 out of 7 layers of the Core 3 displayed values from 1.7 to 4.1 mg/kg, which therefore grouped together and distinctively from the rest (Supplementary Figure 3).

The specific PCA was conducted based on concentrations of “Other elements,” primarily metals and metalloids (Supplementary Figure 4). Iron (Fe), arsenic (As), and manganese (Mn) were the elements which had greatest influence on the PCA; concentrations of Fe and As were much higher in Core 1, while Mn was greatest in Cores 1 and 2, but lower in Core 3. On the other side, zinc (Zn), copper (Cu), and yttrium (Y) were specifically higher in some sublayers of Core 3; however, these are the variables showing less contribution percentage to the patterns shown in the PCA.

### Principal Components Analysis Using All Chemical Properties

All chemical parameters were then analyzed and included in the same PCA (Figure 1). Again, the sublayer 1.3.2 dropped far away from the rest of “the cloud” due to its drastic shift in values on anions concentrations, except PO_4_^3–^. For this reason, the group of the Core 1 showed a very high variance (represented by a big ellipse). However, it is also evident how the remaining variables influenced the separation of the rest of layers groups, with Fe and As concentration pushing for Core 1 group as long as Pb and Br (which was not so clear in the specific PCA for elements) (Figure 1).

Comparison of chemical composition of sediment from the surface and overlaying waters established previously (Korzhenkov et al., 2019) and in this study showed that Al was represented in significantly higher quantities across the gradient, exceeding its concentrations on the surface up to 5–9 times. Concentrations of K and Ti determined in sediment core layers at various depths were >2-fold higher than at the surface, whereas Cr and Mn were present at lower concentrations. Ni, Zn, Ca, As, and Sr had about the same concentrations across samples with few exceptions (e.g., more abundant in some layers). Pb was generally detected in lower quantities than on the surface, however, there were few exceptions. Fe was found in high various quantities in different layers of sediment, comparable with those at the surface (66.7 g/kg) (Supplementary Table 1).

Total carbon and nitrogen were less abundant in deeper layers in comparison to those at the surface (2.8 and 0.3%, respectively) (Supplementary Table 1). C:N ratio was highly variable (0.8–26.7) across the different layers and was not dependent on depth (Supplementary Table 1).

GC-MS analysis of black layers (oily deposits) from Parys Mt acidic stream sediment identified hydrocarbons, specifically unbranched alkanes with C_17_ being the most abundant type.

## Microbial Content

### Taxonomic Composition of Microbial Communities in Sediment Layers

#### Archaea

**FIGURE 2:**
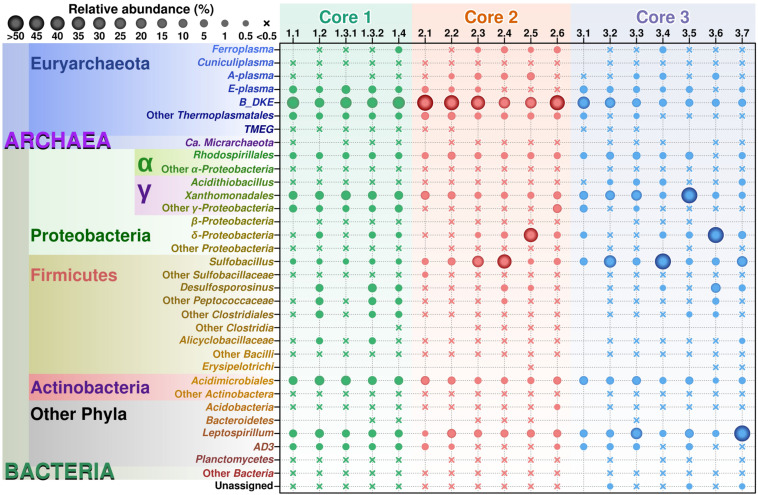
Relative abundance of various taxonomic groups in Parys Mt sediments. OTUs found after analysis of the sequencing results were grouped by lineage on those most abundant taxons, from lowest to higher levels, with genus as the basic clustering level where possible. The final table was generated with 30 taxonomic groups. From this table, a balls diagram was produced showing the relative abundance of these taxonomic groups.

Archaeal sequences were found in all three cores (Figure 2), which is in accordance with previous studies investigating surface sediments (0–3 cm) at this site (Korzhenkov et al., 2019). Across different sediment depths, archaea represented a dominant group, as judged from the total number of reads and numbers of OTUs, particularly in Cores 1 and 2. In Core 3, a very large number (ca. 30%) of archaeal reads were observed in the upper sediment layer; all deeper layers displayed a consistent decrease of archaeal reads (down to 6%) and increase in various bacterial groups.

Archaeal diversity has been mostly restricted to Euryarchaeota (or Thermoplasmatota, according to the GDTB taxonomy^1^), and among those, mainly to the members of the order Thermoplasmatales. In this study, Thermoplasmatales reads were detected in high abundance as follows: (i) in Core 1 it ranged from 49% of the total reads at the surface to 39.5% at a depth of 6–8 cm; (ii) in Core 2 they represented 54.1% of total reads at the surface to 51.5% at a depth of 10–15 cm; (iii) in Core 3 they represented 39.9% at the surface to 6.2% at a depth of 20 cm.

**FIGURE 3:**
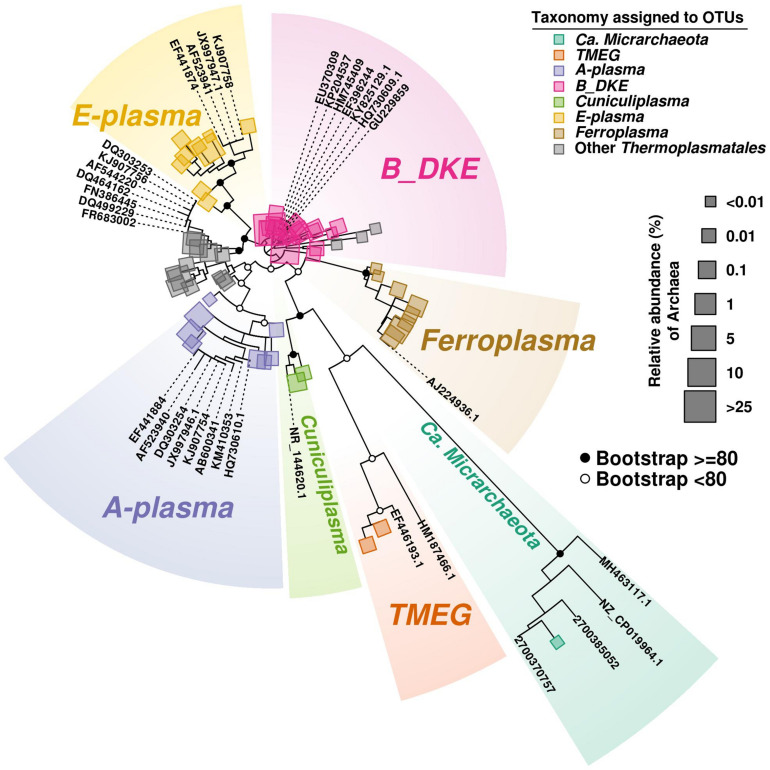
Phylogenetic tree of Archaea. The tree was developed to include the most abundant OTU (>100 reads) sequences found along the three cores. Bootstrap values are shown on main parental nodes, where open dots represent bootstrap values under 80, while closed black dots represent values equal or higher to 80. OTU sequences are represented by colored squares corresponding to their assigned taxonomy (see section “Bioinformatics Analysis in the Materials and Methods”), while size corresponds to their relative abundance (%) relative to the amount of Archaea present. Reference sequences are represented by their accession number on GeneBank or IMG/M system.

Among Thermoplasmatales, the most abundant group of sequences across the depth gradient was affiliated to B_DKE metagenomic assembly. These sequences represented 5–45% of the total with the greatest abundance seen in Core 2. This group has also previously been reported in pyrite mine biofilm (Harz Mountains, Germany) by Krause et al. (2017). These archaea were followed by the “E-plasma” variant which was present in all three cores with varying numbers (0.5–15%) depending upon depth. In addition, within Cores 1 and 2, sequences similar to the phylotype with accession number FR683002 and to other unclassified Thermoplasmatales were detected (<0.5–10%). Reads related with “A-plasma” metagenomic assembly, *Ferroplasma acidiphilum*- (both in quantities <0.5–5%) and *Cuniculiplasma divulgatum*-related (with a relative abundance of <0.5%) organisms were also identified. These phylotypes clustered with known taxonomic clades of archaea or reference organisms, as demonstrated in Figure 3 and Supplementary Table 2. No correlation of relative numbers of these taxonomic groups with sediment depth was seen. However, in the case of “A-plasma”- and *F. acidiphilum*-related organisms, their abundance gradually increased with depth down to the black-colored layer (Figure 2). Maximal numbers of “A-plasma” were observed at 8–10 cm (Core 2), and for *Ferroplasma*-like sequences at 6–8 cm (Core 1), 4–15 cm (Core 2), and 9–11 cm (Core 3). Interestingly, *Ferroplasma* reads were not detected in upper layers of all three cores, and their presence has not previously been reported in any other parts of the Parys Mt ecosystem (Korzhenkov et al., 2019). *Cuniculiplasma* spp. was the lowest-abundance group among other Thermoplasmatales with a relative abundance <0.5% across all layers and depths. These archaea were also earlier shown to only comprise a minor group in the upper sediment/water stream community (Korzhenkov et al., 2019).

In this study, minor quantities (0.1–0.5%) were affiliated with TMEG-related organisms (Terrestrial Miscellaneous Euryarchaeal group, or ambiguous taxa in the class Thermoplasmata, as per the SILVA database v.132). The relative abundance of this group were relatively constant with depth in Core 1, but were mostly detected in the upper sections of Cores 2 and 3. Furthermore, “*Ca*. Micrarchaeota” was present in very low abundance (<0.5%) across almost all sediment depths. Both groups were shown previously to inhabit the uppermost layer of sediments and can also be found in the overlying stream water (Korzhenkov et al., 2019).

All archaea of the order Thermoplasmatales described so far are prominent inhabitants of acidic environments and exhibit a heterotrophic lifestyle, which is reflected in their preferential growth on complex polypeptides (Golyshina, 2011; Golyshina et al., 2016b). *Thermoplasma* was also shown to possess the potential for sulfur-driven respiration with organic carbon as an electron donor (Darland et al., 1970). Furthermore, members of the family Ferroplasmaceae are able to undertake iron oxidation/reduction (Golyshina, 2011). Heterotrophy and iron redox cycling (iron is highly available under oxidative redox conditions and low pH) together with facultatively anaerobic capability are likely present among archaeal components of these sediment communities. The occurrence of Fe (III) reduction in acidic sediments at low oxygen concentration was reported previously (Küsel et al., 2002). Sulfur respiration could potentially be another trait of these archaea. Iron redox cycling and heterotrophy were confirmed experimentally for cultured mesophilic species of *F. acidiphilum* and *C. divulgatum*, respectively (Golyshina et al., 2000, 2016b). However, since the majority of archaea populating this environment are uncultured, their metabolic properties remain to be confirmed.

It should be noted that all these archaeal phylotypes are widely found in a range of acidic environments. Archaea designated as B_DKE were identified in enrichment cultures established with biofilms obtained from a pyrite mine (Harz Mountains, Germany) (Krause et al., 2017). The organism was shown to grow in anaerobic enrichment culture when the medium was supplemented with polypeptides and ferric sulfate; furthermore, the authors suggested that these archaea could undertake ferric iron reduction (Krause et al., 2017). Similar features are highly likely for B_DKE archaea although their physiological properties still need to be confirmed in pure culture. Similar organisms are present in various low-pH environments (Krause et al., 2017). For example, almost identical SSU rRNA gene sequences with accession numbers HQ730609, EU370309, HM745409, and EF396244 were detected in anaerobic sediments and biofilm communities from Rio Tinto (Spain), an extremely acidic, metal-rich stream (Huelva, Spain), and in La-Zarza-Perrunal acid mine effluent (Spain) (Rowe et al., 2007; Gonzalez-Torril et al., 2011; Sánchez-Andrea et al., 2011). Moreover, similar phylotypes were recovered from a low temperature (8.5°C) underground mine at Cae Coch (GU229859, Wales, United Kingdom) (Kimura et al., 2011), and in an acidic geothermal area (35°C) of Copahue (KP204537, Neuquen, Argentina) (Urbieta et al., 2015).

Other most-abundant phylotypes from Parys Mt sediments were clustered with the sequence with the accession number FR683002 from the microbial community of Pb-Zn mine, and also in acid mineral bioleaching systems of Dongxiang copper mine, Yinshan Lead-Zink mine and Yun-Fu pyrite mine (DQ464162; FN386445), all places located in China (Xiao et al., 2008; Tan et al., 2009; Huang et al., 2011). Furthermore, similar sequences were present in macroscopic filaments from Rio Tinto (Spain) (DQ303253, Garcia-Moyano et al., 2007), in cave wall biofilms from the Frasassi cave system, Italy (DQ499229; Macalady et al., 2007), in Iron Mountain AMD system, United States (AF544220; Baker and Banfield, 2003), in thermal and acidophilic biofilms, Mexico (KJ907756; unpublished) and in endolithic microbial community from Rio Tinto basin, Spain (EF441883; unpublished).

Other archaea identified in Parys Mt sediments and still awaiting their isolation are “E-plasma” and “A-plasma” (Baker and Banfield, 2003). Firstly detected in Iron Mountain (United States) metagenomic datasets, these organisms were found in the Parys Mt acidic stream surface sediment, with “E-plasma” as a dominant phylotype (Korzhenkov et al., 2019). Their metabolism was predicted as heterotrophic, which involves iron oxidation/reduction (Yelton et al., 2013). It is worth noting that both are also ubiquitous: they were found, e.g., in macroscopic filaments (DQ303254 and EF441874, correspondingly) (Garcia-Moyano et al., 2007), and in endolithic communities in the Rio Tinto basin (EF441884 and EF441874). The “A-plasma” phylotype was also detected in anaerobic sediments from Rio Tinto (HQ730610; Sánchez-Andrea et al., 2011). Furthermore, 16S rRNA gene amplicon reads of both archaea were found in forested wetland sediment samples influenced by waste coal deposits, United States (AF523940, AF523941; Brofft et al., 2002), in terrestrial subsurface cave systems, Italy (KM410353, AF523941; Hamilton et al., 2015) and in a thermal acidic biofilm, Mexico (KJ907754, KJ907758; unpublished). Additionally, sequences clustering with the “A-plasma” were present in the metagenomic data from a terrestrial acidic spring field, Japan (AB600341; Kato et al., 2011).

In relation to our results, a few points need to be highlighted. Firstly, there is still an extremely small number of archaeal taxa cultured from AMD, in comparison to bacteria. Bacterial acidophilic diversity associated with AMD sites is assigned to more than 13 genera belonging to various phyla (Acidobacteria, Actinobacteria, Firmicutes, Nitrospirae, and Proteobacteria) (Mendez-Garcia et al., 2015; Gavrilov et al., 2019). However, all cultured archaea from similar AMD environments with validly published names are affiliated with the single order, Thermoplasmatales of the phylum Euryarchaeota (genera *Ferroplasma*, *Acidiplasma*, and *Cuniculiplasma*) (Golyshina et al., 2000, 2009, 2016b; Hawkes et al., 2008). Thermophilic crenarchaeon *Metallosphaera prunae* isolated from a uranium mine is the only example of cultured representatives from another higher archaeal taxon (Fuchs et al., 1995). Thus, organisms of the order Thermoplasmatales are considered to be the most successful archaeal colonizers of mining sites, natural or anthropogenic environments with moderate temperatures, benefiting from low pH and oxygen levels. The second important point to consider when assessing sequencing data from similar environments is that the sequences submitted to the databases with the 16S rRNA sequence identity levels below 94% with the reference isolates, are often wrongly qualified as *Thermogymnomonas* spp. or *Thermoplasma* spp., which creates confusion and leads to incorrect interpretation. Importantly, *Thermogymnomonas* or *Thermoplasma* spp. were so far not detected in the low- or moderate-temperature AMD environments.

Other archaea inhabiting Parys Mt sediment belonged to “*Ca*. Micrarchaeota” detected at different depths of the three cores. These sequences showed 98–99% 16S rRNA gene identity levels to organisms from volcanic environments (GQ141757; KJ907762) and from Parys Mt surface parts (Golyshina et al., 2019). 16S rRNA sequence identity of these sediment variants to “*Ca*. Mancarchaeum acidiphilum,” Mia14 was found to be 91.8%.

### Bacteria

Among bacteria, members of the phylum Proteobacteria were most abundant in all cores, comprising on average 26.0 ± 3.5% of the community across all depths. Firmicutes in all layers reached moderate numbers representing 7.2 ± 3.8% of the total community (Figure 2). Other bacterial groups consistently present in all layers were from the phyla Nitrospirae, Actinobacteria, uncultured Chloroflexi (AD3 group), Acidobacteria and others (Figure 2).

No correlation of Proteobacteria distribution with sediment depth was observed. Among Proteobacteria, classes Alphaproteobacteria, Deltaproteobacteria, and Gammaproteobacteria signatures were the most prominent. Gammaproteobacteria were represented mostly by three groups of organisms: the unclassified Gammaproteobacteria, order Xanthomonadales (family Xanthomonadaceae) and the cluster RCP1-48.

Xanthomonadaceae (0.5–45%) were represented mostly by organisms closely related to *Metallibacterium scheffleri*, described as facultatively anaerobic, iron-reducing organisms (Ziegler et al., 2013). In addition, some *Stenotrophomonas* spp. and *Pseudoxanthomonas* spp. were detected. Also, *Acidithiobacillus* spp. -related OTUs, with a rather low sequence identities with type strains (<96–97%) were observed in minor amounts (<0.5–1%). Similarly, low numbers of *Acidithiobacillus* were earlier detected in the surface sediment and water, suggesting that this particular environment is not very favorable to these organisms (Korzhenkov et al., 2019). A possible reason is the extremely low pH (<2), high redox and abundance of Fe (III) in Parys Mt AMD; these factors were previously considered as less advantageous for these organisms (Rawlings et al., 1999). Alphaproteobacteria were detected in quantities from 0.1% to a maximum of 7.3% at all sediment depths. Representatives of Rhodospirillales (family Acetobacteraceae) were seen mostly in OTUs with a very distant phylogenetic position from *Rhodophiala* spp., *Acidisoma* spp., and *Acidisphaera rubrifaciens*, making it challenging to speculate on their metabolism.

Deltaproteobacteria were associated with the order Bdellovibrionales, family Bacteriovoraceae, in which the sequences showed low homology (less than 90%) to described isolates. Patchiness was observed for the vertical distribution of these bacteria. Some increase in numbers of Bacteriovoraceae with depth was observed. Another relatively abundant bacterial phylum was Actinobacteria (<0.5–15%) with OTUs affiliated mostly with Acidimicrobiales. Among them, the sequences similar to *Aciditerrimonas* (95% identity to *Atn. ferriducens*), *Acidimicrobium* (95% identity to *Am. ferrooxidans*) and *Ferrimicrobium acidiphilum* (100%) were detected. *Aciditerrimonas* was described as facultatively anaerobic, heterotrophic and autotrophic organism, able to undertake dissimilatory reduction of ferric iron (Itoh et al., 2011). *Acidimicrobium* and *Ferrimicrobium* are known inhabitants of acidic environments, with the ability to undertake iron oxidation to undergo heterotrophic growth (Mendez-Garcia et al., 2015).

The consistent presence of Nitrospirae (1–20%) was demonstrated at various depths in all cores. Of note, at the depth of 18–20 cm in Core 3, the Nitrospirae OTUs reached 52.1%, with affiliation of all sequences to *Leptospirillum* spp. (Markosyan, 1972; Hippe, 2000; Coram and Rawlings, 2002), represented mostly by *Leptospirillum ferrooxidans*-related organisms and by new species of this genus. All validly published leptospirilli were described as aerobic and autotrophic (ferrous iron oxidizing) organisms (Markosyan, 1972; Hippe, 2000; Coram and Rawlings, 2002).

Firmicutes were found to increase their numbers with depth in Core 1 and varied in numbers in other cores, in line with the physicochemical heterogeneity of the sediments. Among them, the sequences of Sulfobacillus, YNPFFP6 group of Sulfobacillaceae-, *Alicyclobacillus*-, and *Desulfosporosinus-*related bacteria were the most representative OTUs. *Sulfobacillus* and *Alicyclobacillus* spp. are well-known inhabitants of AMD systems with facultatively anaerobic lifestyles and capable of iron oxidation and reduction, oxidation of sulfur compounds and heterotrophic or autotrophic types of carbon assimilation (Mendez-Garcia et al., 2015). Sulfate-reducing *Desulfosporosinus* members were also previously shown to inhabit AMD sediments (Alazard et al., 2010; Sánchez-Andrea et al., 2015). Firmicutes were found to be highly represented in the black-colored layers, reaching proportionally high numbers of 30–50% of total reads. Of note, at a depth of 9–11 cm in Core 3, Firmicutes represented 75.2% of the total reads. The majority of OTUs found were either *Sulfobacillus-* and *Alicyclobacillus*-related sequences, only distantly affiliated to the species with the established taxonomy. Other Firmicutes belonged to *Desulfosporosinus* and other bacteria of the family Peptococcaceae (Clostridiales). Moreover, sequences distantly related to other families of the order Clostridiales were identified in the sequencing data of “black layers.” During the sampling, while inserting sampling corers into the sediments and reaching the “black horizon,” we observed the development on the water surface of a thin hydrophobic film, highly likely, of hydrocarbons. We measured hydrocarbons in two selected samples of “black layers” and identified the *n*-heptadecane as a major component (19 and 43 mg/kg). This compound is known to be the most abundant product in cyanobacteria, but can also hypothetically be formed from fatty acids through reactions catalyzed by reductases and decarboxylases (Kang and Nielsen, 2017). Whatever the origin, this compound can be metabolized by acidophilic bacteria, including *Sulfobacillus* spp., as demonstrated previously (Hamamura et al., 2005; Ivanova et al., 2013).

Across the depths, other bacteria were represented by uncultured Chloroflexi (AD3 group/JG 37-AG-4) in numbers between 0.5–1% for Cores 2 and 3 and ca. 5% within Core 1. The metabolic features of these organisms previously detected in acidic ecosystems, remain unknown (Gavrilov et al., 2019).

**FIGURE 4:**
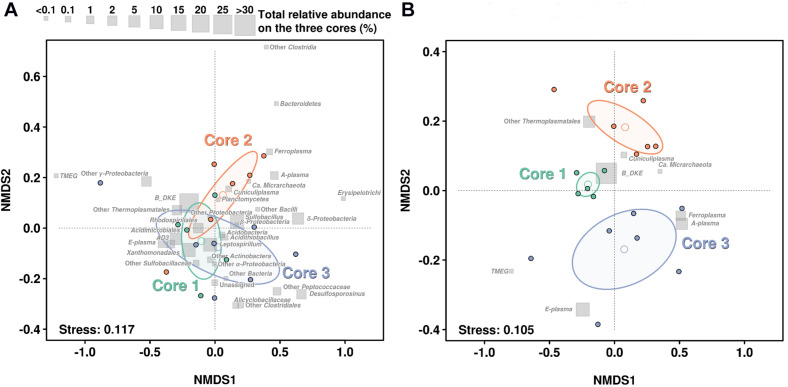
NMDS based on the taxonomic profiles in each sediment core. **(A)** NMDS regarding the distribution of the whole community. **(B)** NMDS regarding only distribution of *Archaea*. Gray squares show the relative abundance of each taxonomic group in all layers on the three cores. Open dots show the mean of each layer while ellipse lines are based on the variance observed among each group of layers on each core. Stress level of analysis return a value of 0.118 and 0.108, which is considered a good or very good model adjustment over the 2D plane.

In order to assess how the abundance profiles differs in the three cores, a non-metric multidimensional scaling (NMDS) was performed, using Bray–Curtis distances (Figure 4). The NMDS of the whole community suggests that the most abundant groups were in general not defining very well the differences over the three cores, hence these groups are mostly concentrated close to the center of the diagram. Additionally, NMDS results emphasized that microbial community stability decreases with depth. So, all samples from Core 1 kept a more similar taxonomic distribution profile than Cores 2 and 3 (see Figure 4). This can also be observed in their ellipse ranges, based on layers variance. Finally, separation among samples seems to be the result of less-abundant taxa, especially in Core 3. For instance, TMEG was detected at very low quantities in all samples, however, the layer 3.1 showed the biggest relative abundance (0.336%) which is about 12-fold higher than the average of TMEG numbers (0.027%). This was also the case with *Sulfobacillus*, which was especially abundant in layer 3.4 (>70%) (Figure 4A).

If we focus on the NMDS representation for Archaea, we can see a very large difference in the distribution. Samples from the Core 1 clustered very compactly showing a very similar distribution of all archaeal groups. In contrast, samples from Core 3 showed a large amount of scatter and largest variance on their ellipse (Figure 4B).

### Correlation Analysis Between Microbial Diversity and Chemical Properties

**FIGURE 5:**
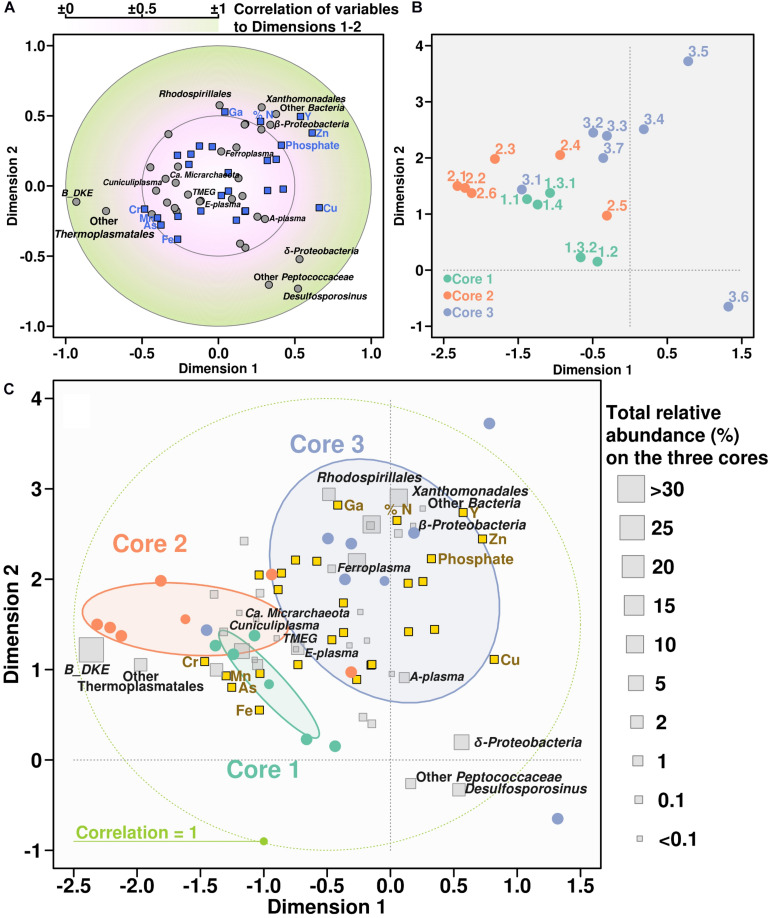
Canonical correlation analysis between chemical variables and microbial community. Panel showing the CCorA among taxonomy distribution, chemical parameters, and the samples representation over the canonical variates. Top panels are separate representations of variables **(A)** and samples **(B)**. Below, same both representations overlapped adding the relative abundance of each taxonomic group along the whole core **(C)**.

Canonical correlation analysis was used to demonstrate the relationship between chemical properties and microbial community composition (in this case, treating microbial groups as variables). According to the CCorA, B_DKE phylotype and also other Thermoplasmatales were the groups with highest correlation with chemical variables, specifically to As, Fe, Cr, and Mn, in comparison with bacteria (Figure 5). All Thermoplasmatales phylotypes and ‘*Ca.* Mancarchaeum acidiphilum’ possess a high genomic potential for metal resistance, as suggested previously in acidophiles (Dopson and Holmes, 2014). Thus, metallochaperones, heavy metal reductases, mercury (II) reductases, CopP type ATPases, arsenic efflux pump-related proteins (ArsA, ArsB, and ArsR) were found in the genomic data of reference organisms (Supplementary Table 3). Genes encoding these proteins were shown previously to be often located on “defense” genomic islands (Golyshina et al., 2016a, 2017).

### Comparison With Other Acidic Sediments

In comparison to other AMD sediments, this particular system is characterized by positive redox potential and relatively low pH (1.7–2.5). The high abundance of archaea shown in this study seems different from earlier analyses due to a lower redox and higher pH values in the latter (Sánchez-Andrea et al., 2011, 2012; Sun et al., 2015). However, Parys Mt and Rio Tinto sediment archaeal phylotypes were found to be similar, supporting the prediction of the versatility of uncultured Thermoplasmatales in relation to the oxygen tolerance and pointing at their potential facultative anaerobic lifestyle. As in the present study, archaea of the order Thermoplasmatales were reported independently of sampling depth and spot at the Rio Tinto mine site (Sánchez-Andrea et al., 2011, 2012).

Diverse archaeal sequences were earlier revealed in the arsenic-rich creek sediment of Carnoules Mine, France (Volant et al., 2012). Archaea (Thermoplasmatales/Euryarchaeota together with Thaumarchaeota) were suggested to be important contributors to carbon and nitrogen cycles in microniches within the sediment. No overlap in archaeal phylotypes from Parys Mt and Carnoules Mine sediments could be observed, while the latter hosted archaea very distantly related with all cultured Thermoplasmatales (Volant et al., 2012). However, a relatively high similarity (about 97–98% SSU rRNA gene sequence identity) was recorded for reads from Carnoules Mine and Los Rueldos biofilm communities (Méndez-García et al., 2014).

The bacterial component in Carnoules Mine included members of genera *Gallionella*, *Thiomonas*, *Acidithiobacillus*, and *Acidiphilium*, all of which are indicative to pH values higher than in Parys Mt sediment (Bruneel et al., 2011).

Low pH favors the presence of other extremely acidophilic microorganisms, e.g., *Leptospirillum* spp. in the sediment samples. These organisms were shown to be completely absent in anoxic and higher pH sediments of Rio Tinto (Sánchez-Andrea et al., 2011, 2012). Other bacterial groups were found to be rather typical and characteristic for AMD sediments. Probably the lack of Bacteroidetes could be noted as a discrepancy in this context, because of the oxic conditions being inhibitory to the acidophilic members of this phylum. Other bacteria presented in large quantities in sedimental microniches, such as Gammaproteobacteria (*Acidibacter ferrireducens*, *M. scheffleri* and RCP1-48 group) together with Actinobacteria (*Aciditerrimonas*, *Acidimicrobium*, and *Ferrimicrobium)* point at the importance of iron metabolism in this ecosystem. Furthermore, their involvement in heterotrophic and autotrophic loops of the carbon cycle Parys Mt sediment is supported by presence of these very phylotypes. Apart from these microorganisms, Sulfobacillaceae and Alicyclobacillaceae families might take part in carbon and iron transformations in Parys Mt, which was also found in AMD sediments in other locations (Sánchez-Andrea et al., 2011, 2012).

Interestingly, the high abundance of particular archaeal taxa of the order Thermoplasmatales in Parys Mt sediments occurred across all samples, independently of variations in pH, Eh, and depth. However, once again, this group of organisms and overall the archaeal members of low-pH environments are significantly lagging behind their much better metabolically characterized bacterial counterparts. This is primarily associated with the difficulties of cultivation of archaea, for which (i.e., for the vast majority of members of Thermoplasmatales) only genome-informed predictions of metabolic traits are available. We suggest that the lifestyles and ecological roles of archaea in sediments of Parys Mt are based on the degradation of organic compounds from primary producers and, e.g., scavenging protein/polypeptide-rich biomass detritus and on the inorganic iron and sulfur compounds conversions. Further research is needed to understand the contribution of particular archaeal organisms inhabiting this ecosystem.

## Conclusion

The environmental conditions in Parys Mt sediment underlying the AMD stream determined the make-up of the microbial community with a large proportion of Thermoplasmatales archaea, which were abundant at various depths and sediment layers. Bacterial community members, generally less abundant than archaea, varied in numbers more significantly across different depths, their taxonomic affiliations pointed at their involvement in metabolism of carbon, iron, and sulfur elements. The decisive factors favoring high archaeal numbers are the low pH (1.7–2.4), the positive redox potential, availability of carbon sources (polypeptides-rich detritus/dead biomass), electron donors (ferrous iron, sulfur compounds, or carbon) and acceptors (ferric iron and oxygen). Importantly, a positive relationship was identified between Fe, As, Cr, and Mn contents and archaeal abundance, which points toward a strong tolerance of Thermoplasmatales to the high concentrations of dissolved metals and metalloids. Significant numbers of archaea in AMD sediments and the ubiquity of similar systems on our planet suggest Thermoplasmatales may have a greater impact on the global carbon, sulfur, and iron cycling than currently assumed. Further efforts are required to investigate their roles in the environment through cultivation and omics-driven analyses of their physiology and metabolism.

## Data Availability Statement

The datasets presented in this study can be found in online repositories. The names of the repository/repositories and accession number(s) can be found in the article/Supplementary Material.

## Author Contributions

PG and OG conception of the work. PG, MD, GW, and FB undertook the field and laboratory work. FB, SW and DJ undertook the chemical analysis MD, EL and RB acquisition of the data for the work. RB, EL, ST, MY, PG, and OG interpretation of the data for the work. OG, RB, and PG drafted the manuscript with further contribution from all authors. All authors contributed to the article and approved the submitted version.

## Conflict of Interest

The authors declare that the research was conducted in the absence of any commercial or financial relationships that could be construed as a potential conflict of interest.

## Footnotes

1 https://gtdb.ecogenomic.org

## References

[B1] Alazard D. Joseph M. Battaglia-Brunet F. Cayol J. L. Ollivier B. (2010). Desulfosporosinus acidiphilus sp. nov.: a moderately acidophilic sulfate-reducing bacterium isolated from acid mining drainage sediments: new taxa: firmicutes (Class *Clostridia*, Order *Clostridiales*, Family *Peptococcaceae*). *Extremophiles* 14 305–312. 10.1007/s00792-010-0309-4 20358236

[B2] Baker B. J. Banfield J. F. (2003). Microbial communities in acid mine drainage. *FEMS Microbiol. Ecol.* 44 139–152. 10.1016/s0168-6496(03)00028-x 19719632

[B3] Ball D. F. (1964). Loss-on-ignition as an estimate of organic matter and organic carbon in non-calcareous soil. *J. Soil Sci.* 15 84–92. 10.1111/j.1365-2389.1964.tb00247.x

[B4] Brofft J. E. McArthur J. V. Shimkets L. J. (2002). Recovery of novel bacterial diversity from a forested wetland impacted by reject coal. *Environ. Microbiol.* 4 764–769. 10.1046/j.1462-2920.2002.00337.x 12460285

[B5] Bruneel O. Volant A. Gallien S. Chaumande B. Casiot C. Carapito C. (2011). Characterization of the active bacterial community involved in natural attenuation processes in arsenic-rich creek sediments. *Microb. Ecol.* 61 793–810. 10.1007/s00248-011-9808-9 21318282

[B6] Camacho C. Coulouris G. Avagyan V. Ma N. Papadopoulos J. Bealer K. (2008). BLAST+: architecture and applications. *BMC Bioinformatics* 10:421. 10.1186/1471-2105-10-421 20003500 PMC2803857

[B7] Capella-Gutierrez S. Silla-Martinez J. M. Gabaldon T. (2009). trimAl: a tool for automated alignment trimming in large-scale phylogenetic analyses. *Bioinformatics* 25 1972–1973. 10.1093/bioinformatics/btp348 19505945 PMC2712344

[B8] Coram N. J. Rawlings D. E. (2002). Molecular relationship between two groups of the genus *Leptospirillum* and the finding that *Leptospirillum ferriphilum* sp. nov. dominates South African commercial biooxidation tanks that operate at 40 °C. *Appl. Environ. Microbiol.* 68 838–845. 10.1128/AEM.68.2.838-845.2002 11823226 PMC126727

[B9] Darland G. Brock T. D. Samsonoff W. Conti S. F. (1970). A thermophilic, acidophilic mycoplasma isolated from a coal refuse pile. *Science* 170 1416–1418. 10.1126/science.170.3965.1416 5481857

[B10] Dopson M. Holmes D. S. (2014). Metal resistance in acidophilic microorganisms and its significance for biotechnologies. *Appl. Microbiol. Biotechnol.* 98 8133–8144. 10.1007/s00253-014-5982-2 25104030

[B11] Fadrosh D. W. Ma B. Gajer P. Sengamalay N. Ott S. Brotman R. M. (2014). An improved dual-indexing approach for multiplexed 16S rRNA gene sequencing on the Illumina MiSeq platform. *Microbiome* 2:6. 10.1186/2049-2618-2-6 24558975 PMC3940169

[B12] Fuchs T. Huber H. Teiner K. Burggraf K. Stetter K. O. (1995). *Metallosphaera prunae*, sp. nov., a novel metal-mobilizing, thermo-acidophilic *archaeum*, isolated from a uranium mine in Germany. *Syst. Appl. Microbiol.* 18 560–566. 10.1016/S0723-2020(11)80416-9

[B13] Garcia-Moyano A. Gonzalez-Toril E. Aguilera A. Amils R. (2007). Prokaryotic community composition and ecology of floating macroscopic filaments from an extreme acidic environment. Rio Tinto(SW, Spain). *Syst. Appl. Microbiol.* 30 601–614. 10.1016/j.syapm.2007.08.002 17950555

[B14] Gavrilov S. N. Korzhenkov A. A. Kublanov I. V. Bargiela R. Zamana L. V. Popova A. A. (2019). Microbial communities of polymetallic deposits”acidic ecosystems of continental climatic zone with high temperature contrasts. *Front. Microbiol.* 10:1573. 10.3389/fmicb.2019.01573 31379766 PMC6650587

[B15] Golyshina O. V. (2011). Environmental, biogeographic, and biochemical patterns of archaea of the family *Ferroplasmaceae*. *Appl. Environ. Microbiol.* 77 5071–5078. 10.1128/AEM.00726-11 21685165 PMC3147448

[B16] Golyshina O. V. Bargiela R. Toshchakov S. V. Chernyh N. A. Ramayah S. Korzhenkov A. A. (2019). Diversity of “*Ca*. Micrarchaeota” in two distinct types of acidic environments and their associations with Thermoplasmatales. *Genes* 10:461. 10.3390/genes10060461 31208064 PMC6627985

[B17] Golyshina O. V. Kublanov I. V. Tran H. Korzhenkov A. A. Lünsdorf H. Nechitaylo T. Y. (2016a). Biology of archaea from a novel family *Cuniculiplasmataceae* (*Thermoplasmata*) ubiquitous in hyperacidic environments. *Sci. Rep.* 6:9034. 10.1038/srep39034 27966672 PMC5155288

[B18] Golyshina O. V. Lünsdorf H. Kublanov I. V. Goldenstein N. I. Hinrichs K. U. Golyshin P. N. (2016b). The novel extremely acidophilic, cell-wall-deficient archaeon *Cuniculiplasma divulgatum* gen. nov., sp. nov. represents a new family, *Cuniculiplasmataceae* fam. nov., of the order Thermoplasmatales. *Int. J. Syst. Evol. Microbiol.* 66 332–340. 10.1099/ijsem.0.000725 26518885 PMC4806541

[B19] Golyshina O. V. Pivovarova T. A. Karavaiko G. I. Kondratéva T. F. Moore E. R. Abraham W. R. (2000). *Ferroplasma acidiphilum* gen. nov., sp. nov., an acidophilic, autotrophic, ferrous-iron-oxidizing, cell-wall-lacking, mesophilic member of the *Ferroplasmaceae* fam. nov., comprising a distinct lineage of the Archaea. *Int. J. Syst. Evol. Microbiol.* 50 997–1006. 10.1099/00207713-50-3-997 10843038

[B20] Golyshina O. V. Tran H. Reva O. N. Lemak S. Yakunin A. F. Goesmann A. (2017). Metabolic and evolutionary patterns in the extremely acidophilic archaeon *Ferroplasma acidiphilum* YT. *Sci. Rep.* 7:3682. 10.1038/s41598-017-03904-5 28623373 PMC5473848

[B21] Golyshina O. V. Yakimov M. M. Lünsdorf H. Ferrer M. Nimtz M. Timmis K. N. (2009). *Acidiplasma aeolicum* gen. nov., sp. nov., a euryarchaeon of the family *Ferroplasmaceae* isolated from a hydrothermal pool, and transfer of *Ferroplasma cupricumulans* to *Acidiplasma cupricumulans* comb. nov. *Int. J. Syst. Evol. Microbiol.* 59 2815–2823. 10.1099/ijs.0.009639-0 19628615

[B22] Gonzalez-Torril E. Aguilera A. Souza-Egipsy V. Lopez Pamo E. Sanchez-Espana J. Amils R. (2011). Geomicrobiology of La Zarza-Perrunal acid mine effluent (Iberian Pyritic Belt, Spain). *Appl. Environ. Microbiol.* 77 2685–2694. 10.1128/AEM.02459-10 21357431 PMC3126378

[B23] Hamamura N. Olson S. H. Ward D. M. Inskeep W. P. (2005). Diversity and functional analysis of bacterial communities associated with natural hydrocarbon seeps in acidic soils at Rainbow Springs, Yellowstone National Park. *Appl. Environ. Microbiol.* 71 5943–5950. 10.1128/AEM.71.10.5943-5950.2005 16204508 PMC1265959

[B24] Hamilton T. L. Jones D. S. Schaperdoth I. Macalady J. L. (2015). Metagenomic insights into S(0) precipitation in a terrestrial subsurface lithoautotrophic ecosystem. *Front. Microbiol.* 5:756. 10.3389/fmicb.2014.00756 25620962 PMC4288042

[B25] Hawkes R. B. Franzmann P. D. O’Hara G. Plumb J. J. (2008). Ferroplasma cupricumulans sp. nov. In List of New Names and New Combinations Previously Effectively, but not Validly, Published, Validation List no. 119. *Int. J. Syst. Evol. Microbiol.* 58 1–2. 18175670 10.1099/ijs.0.65794-0

[B26] Hippe H. (2000). Leptospirillium gen. nov (ex Markoysan 1972), nom. rev., including *Leptospirillium ferrooxidans* sp. nov. (ex Markoysan 1972), nom. rev. and *Leptospirillium thermoferrooxidans* sp. nov. (Golovacheva et al. 1992) *Int. J. Syst. Evol. Microbiol.* 50 501–503. 10.1099/00207713-50-2-501 10758852

[B27] Huang L. N. Zhou W. H. Hallberg K. B. Wan C. Y. Li J. Shu W. S. (2011). Spatial and temporal analysis of the microbial community in the tailings of aPb-Zn mine generating acidic drainage. *Appl. Environ. Microbiol.* 77 5540–5544. 10.1128/AEM.02458-10 21705549 PMC3147485

[B28] Itoh T. Yamanoi K. Kudo T. Ohkuma M. Takashina T. (2011). *Aciditerrimonas ferrireducens* gen. nov., sp. nov., an iron-reducing thermoacidophilic actinobacterium isolated from a solfataric field. *Int. J. Syst. Evol. Microbiol.* 61 1281–1285. 10.1099/ijs.0.023044-0 20639230

[B29] Ivanova A. E. Kizilova A. K. Kanat’eva A. Y. Kravchenko I. K. Kurganov A. A. Belyaev S. S. (2013). A hydrocarbon-oxidizing acidophilic thermotolerant bacterial association from sulfur blocks. *Microbiology* 82 482–489. 10.1134/s0026261713040048 25509383

[B30] Johnson D. B. (2012). Geomicrobiology of extremely acidic subsurface environments. *FEMS Microbiol. Ecol.* 81 2–12. 10.1111/j.1574-6941.2011.01293.x 22224750

[B31] Johnson D. B. Hallberg K. B. Hedrich S. (2014). Uncovering a microbial enigma: isolation and characterization of the streamer-generating, iron-oxidizing, acidophilic bacterium “*Ferrovum myxofaciens*”. *Appl. Environ. Microbiol.* 80 672–680. 10.1128/AEM.03230-13 24242243 PMC3911105

[B32] Jones R. M. Johnson D. B. (2015). *Acidithrix ferrooxidans* gen. nov., sp. nov.; a filamentous and obligately heterotrophic, acidophilic member of the Actinobacteria that catalyzes dissimilatory oxido-reduction of iron. *Res. Microbiol.* 166 111–120. 10.1016/j.resmic.2015.01.003 25638020

[B33] Kang M. Nielsen J. (2017). Biobased production of alkanes and alkenes through metabolic engineering of microorganisms. *J. Ind. Microbiol. Biotechnol.* 44 613–622. 10.1007/s10295-016-1814-y 27565672 PMC5408033

[B34] Kato S. Itoh T. Yamagashi A. (2011). Archaeal diversity in a terrestrial acidic spring field revealed by a novel PCR primer targeting archaeal 16S rRNA genes. *FEMS Microbiol. Lett.* 319 34–43. 10.1111/j.1574-6968.2011.02267.x 21410512

[B35] Katoh K. Standley D. M. (2013). mafft multiple sequence alignment software Version 7: improvements in performance and usability. *Mol. Biol. Evolut.* 30 772–780. 10.1093/molbev/mst010 23329690 PMC3603318

[B36] Kimura S. Bryan C. G. Hallberg K. B. Johnson D. B. (2011). Biodiversity and geochemistry of an extremely acidic, low-temperature subterranean environment sustained by chemolithotrophy. *Environ. Microbiol.* 13 2092–2104. 10.1111/j.1462-2920.2011.02434.x 21382147

[B37] Kock D. Schippers A. (2008). Quantitative microbial community analysis of three different sulfidic mine tailing dumps generating acid mine drainage. *Appl. Environ. Microbiol.* 74 5211–5219. 10.1128/aem.00649-08 18586975 PMC2519280

[B38] Korzhenkov A. A. Toshchakov S. V. Bargiela R. Gibbard H. Ferrer M. Teplyuk A. V. (2019). Archaea dominate the microbial community in an ecosystem with low-to-moderate temperature and extreme acidity. *Microbiome* 7:11. 10.1186/s40168-019-0623-8 30691532 PMC6350386

[B39] Krause S. Bremges A. Munch P. C. McHardy A. C. Gescher J. (2017). Characterisation of a stable laboratory co-culture of acidophilic nanoorganisms. *Sci. Rep.* 7:3289. 10.1038/s41598-017-03315-6 PMC5468238 28607432

[B40] Küsel K. Roth U. Drake H. L. (2002). Microbial reduction of Fe(III) in the presence of oxygen under low pH conditions. *Environ. Microbiol.* 4 414–421. 10.1046/j.1462-2920.2002.00314.x 12123477

[B41] Lukhele T. Selvarajan R. Nyoni H. Mamba B. B. Msagati T. A. M. (2019). Diversity and functional profile of bacterial communities at Lancaster acid mine drainage dam, South Africa as revealed by 16S rRNA gene high-throughput sequencing analysis. *Extremophiles* 23 719–734. 10.1007/s00792-019-01130-7 31520125

[B42] Macalady J. L. Jones D. S. Lyon E. H. (2007). Extremely acidic, pendulous cave wall biofilms from the Frasassi cave system. Italy. *Environ. Microbiol.* 9 1402–1414. 10.1111/j.1462-2920.2007.01256.x 17504478

[B43] Markosyan G. E. (1972). A new iron-oxidizing bacterium *Leptospirillum ferrooxidans* nov. gen. nov. sp. *Biol. J. Armenia* 25 26–29.

[B44] Méndez-García C. Mesa V. Sprenger R. R. Richter M. Diez M. S. Solano J. (2014). Microbial stratification in low pH oxic and suboxic macroscopic growths along an acid mine drainage. *ISME J.* 8 1259–1274. 10.1038/ismej.2013.242 24430486 PMC4030236

[B45] Mendez-Garcia C. Pelaez A. I. Mesa V. Sanchez J. Golyshina O. V. Ferrer M. (2015). Microbial diversity and metabolic networks in acid mine drainage habitats. *Front. Microbiol.* 6:475. 10.3389/fmicb.2015.00475 26074887 PMC4448039

[B46] Oksanen J. Blanchet F. G. Kindt R. Legendre P. Minchin P. R. O’Hara R. B. (2019). *Vegan: Community Ecology Package. R package version 2.5–6.*

[B47] R Development Core Team (2008). *R: A Language and Environment for Statistical Computing.* Vienna: R Foundation for Statistical Computing.

[B48] Rawlings D. E. Tributsch H. Hansford G. S. (1999). Reasons why ‘Leptospirillum’-like species rather than *Thiobacillus ferrooxidans* are the dominant iron-oxidizing bacteria in many commercial processes for the biooxidation of pyrite and related ores. *Microbiology* 145 5–13. 10.1099/13500872-145-1-5 10206710

[B49] Rowe O. F. Sanchez-Espana J. Hallberg K. B. Johnson D. B. (2007). Microbial communities and geochemical dynamics in an extremely acidic, metal-rich stream at an abandoned sulfide mine (Huelva, Spain) underpinned by two functional primary production systems. *Environ. Microbiol.* 9 1761–1771. 10.1111/j.1462-2920.2007.01294.x 17564609

[B50] Sánchez-Andrea I. Knittel K. Amann R. Amils R. Sanz J. L. (2012). Quantification of Tinto River sediment microbial communities: importance of sulfate-reducing bacteria and their role in attenuating acid mine drainage. *Appl. Environ. Microbiol.* 78 4638–4645. 10.1128/aem.00848-12 22544246 PMC3370487

[B51] Sánchez-Andrea I. Rodríguez N. Amils R. Sanz J. L. (2011). Microbial diversity in anaerobic sediments at Rio Tinto, a naturally acidic environment with a high heavy metal content. *Appl. Environ. Microbiol.* 77 6085–6093. 10.1128/aem.00654-11 21724883 PMC3165421

[B52] Sánchez-Andrea I. Stams A. J. Hedrich S. Ňancucheo I. Johnson D. B. (2015). *Desulfosporosinus acididurans* sp. nov.: an acidophilic sulfate-reducing bacterium isolated from acidic sediments. *Extremophiles* 19 39–47. 10.1007/s00792-014-0701-6 25370366

[B53] Sun W. Xiao T. Sun M. Dong Y. Ning Z. Xiao E. (2015). Diversity of the sediment microbial community in the Aha Watershed (Southwest China) in response to acid mine drainage pollution gradients. *Appl. Environ. Microbiol.* 81 4874–4884. 10.1128/aem.00935-15 25979900 PMC4495191

[B54] Tan G. L. Shu W. S. Zhou W. H. Li X. L. Lan C. Y. Huang L. N. (2009). Seasonal and spatial variations in microbial community structure and diversity in the acid stream draining across an ongoing surface mining site. *FEMS Microbiol. Ecol.* 70 121–129. 10.1111/j.1574-6941.2009.00744.x 19678846

[B55] Urbieta M. S. Gonzalez-Toril E. Bazan A. A. Giaveno M. A. Donati E. (2015). Comparison of the microbial communities of hot springs waters and the microbial biofilms in the acidic geothermal area of Copahue (Neuquén, Argentina). *Extremophiles* 19 437–450. 10.1007/s00792-015-0729-2 25605537

[B56] USEPA (2007). *SW-846 Test Method 3550C: Ultrasonic Extraction.* Washington DC: US Environmental Protection Agency.

[B57] Volant A. Desoeuvre A. Casiot C. Lauga B. Delpoux S. Morin G. (2012). Archaeal diversity: temporal variation in the arsenic-rich creek sediments of Carnoulès Mine, France. *Extremophiles* 16 645–657. 10.1007/s00792-012-0466-8 22714283

[B58] Wakelin S. A. Anand R. R. Reith F. Gregg A. L. Noble R. R. P. Goldfarb K. C. (2012). Bacterial communities associated with a mineral weathering profile at a sulphidic mine tailings dump in arid Western Australia. *FEMS Microbiol. Ecol.* 79 298–311. 10.1111/j.1574-6941.2011.01215.x 22092956

[B59] Xiao S. Xie X. Liu J. He Z. Hu Z. (2008). Compositions and structures of archaeal communities in acid mineral bioleaching systems of Dongxiang Copper Mine and Yinshan Lead-Zinc Mine. *China. Curr. Microbiol.* 57 239–244. 10.1007/s00284-008-9183-z 18592308

[B60] Yelton A. P. Comolli L. R. Justice N. B. Castelle C. Denef V. J. Thomas B. C. (2013). Comparative genomics in acid mine drainage biofilm communities reveals metabolic and structural differentiation of co-occurring archaea. *BMC Genomics* 14:485. 10.1186/1471-2164-14-485 23865623 PMC3750248

[B61] Zhang X. Tang S. Wang M. Sun W. Xie Y. Peng H. (2019). Acid mine drainage affects the diversity and metal resistance gene profile of sediment bacterial community along a river. *Chemosphere* 217 790–799. 10.1016/j.chemosphere.2018.10.210 30453276

[B62] Ziegler S. Waidner B. Itoh T. Schumann P. Spring S. Gescher J. (2013). *Metallibacterium scheffleri* gen. nov., sp. nov., an alkalinizing gammaproteobacterium isolated from an acidic biofilm. *Int. J. Syst. Evol. Microbiol.* 63 1499–1504. 10.1099/ijs.0.042986-0 22863988

