# Corrigendum: High Representation of Archaea Across All Depths in Oxic and Low-pH Sediment Layers Underlying an Acidic Stream

**DOI:** 10.3389/fmicb.2021.633015

**Published:** 2021-01-29

**Authors:** Marco A. Distaso, Rafael Bargiela, Francesca L. Brailsford, Gwion B. Williams, Samuel Wright, Evgenii A. Lunev, Stepan V. Toshchakov, Michail M. Yakimov, David L. Jones, Peter N. Golyshin, Olga V. Golyshina

**Affiliations:** ^1^School of Natural Sciences, Bangor University, Bangor, United Kingdom; ^2^Centre for Environmental Biotechnology, Bangor University, Bangor, United Kingdom; ^3^School of Agriculture and Environment, The University of Western Australia, Perth, WA, Australia; ^4^Institute of Living Systems, Immanuel Kant Baltic Federal University, Kaliningrad, Russia; ^5^National Research Centre “Kurchatov Institute”, Moscow, Russia; ^6^Institute for Biological Resources and Marine Biotechnology, CNR, Messina, Italy

**Keywords:** acidophilic archaea and bacteria, Thermoplasmatales, “*Candidatus* Micrarchaeota”, unclassified Euryarchaeota/Terrestrial Miscellaneous Euryarchaeotal Group, acid mine drainage systems, mine-impacted environments, sediment microbiome

In the original article, there was a mistake. The incorrect Figure 1 was published. The caption is correct as published. The correct Figure 1 appears below.

**Figure 1:**
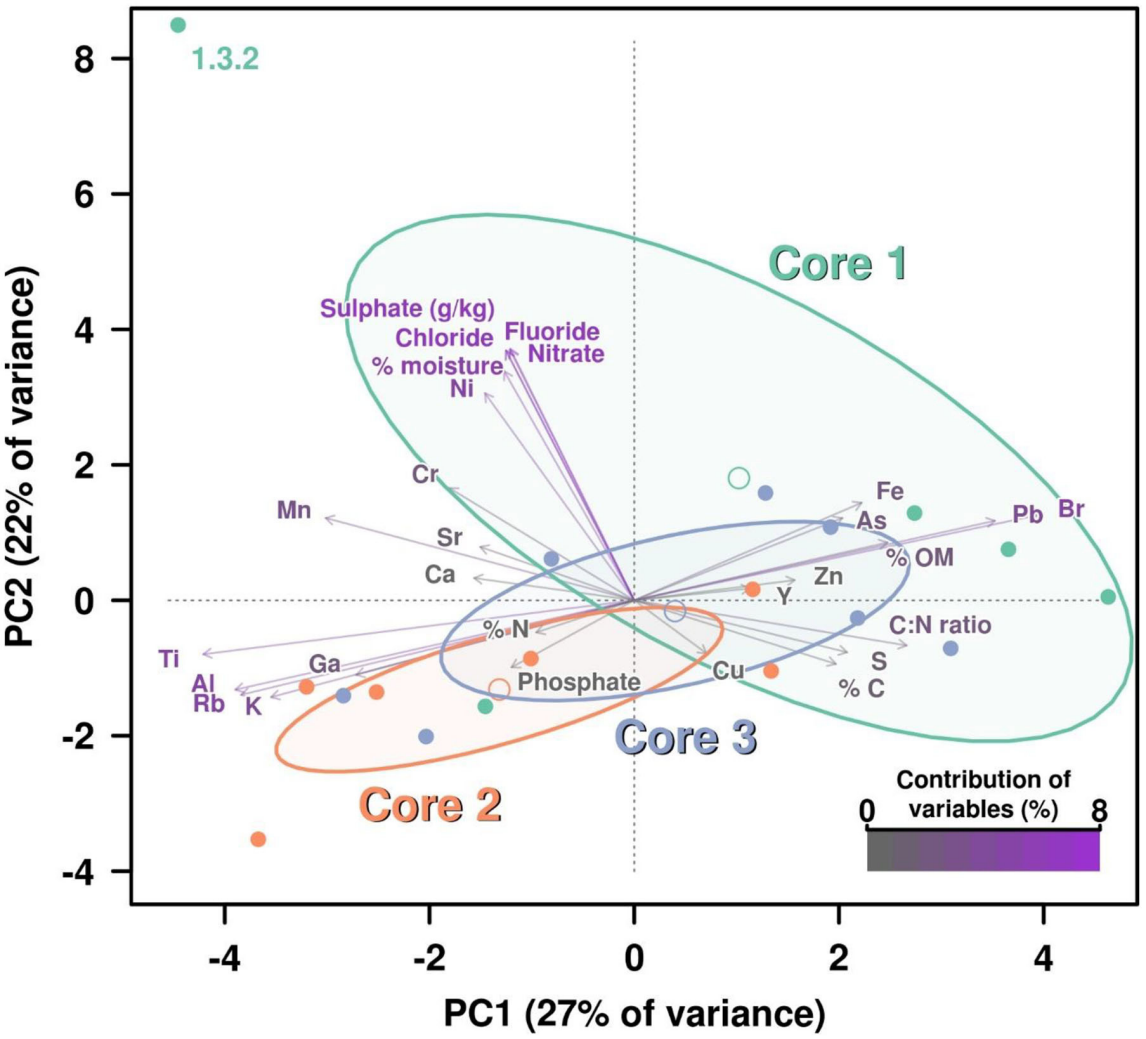
PCA including all chemical parameters analysis by principal components analysis (PCA) of the influence of all chemical properties measured on the three cores. Contribution of each variable (chemical properties) to this graphical representation is shown by a color key from medium gray (less contribution) to violet (highest contribution). Ellipses and open dots represent the variance and mean for each core, respectively. Anion concentrations are showing the highest percentages of contribution due to the higher figures on these values for measured on layer 1.3.2, which is disrupting the variance (ellipse) corresponding to Core 1.

The authors apologize for this error and state that this does not change the scientific conclusions of the article in any way. The original article has been updated.

