# Supplementary material for: High Representation of Archaea Across All Depths in Oxic and Low-pH Sediment Layers Underlying an Acidic Stream
Source: Front Microbiol. 2020 Nov 17;11:576520. doi: 10.3389/fmicb.2020.576520 (PMC7716880; doi:10.3389/fmicb.2020.576520)
Supplement: Supplementary file 1 [file Data_Sheet_1.docx]

**Supplementary Material**

High representation of archaea across all depths in oxic and low-pH sediment layers underlying an acidic stream

Distaso et al.


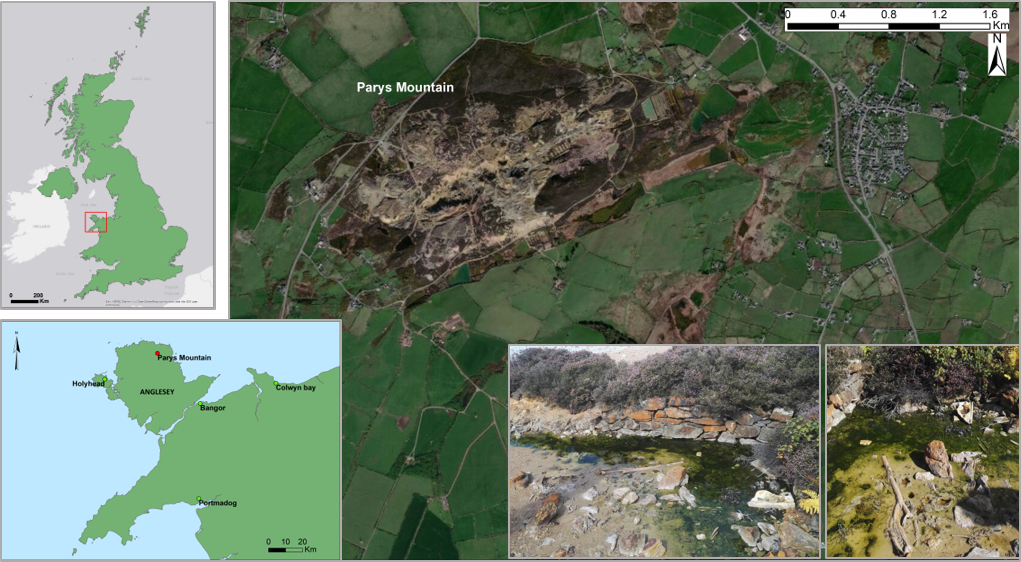


**Figure S1.** Location of Parys Mountain on the island of Anglesey (North Wales, UK), and bottom right inset, photos showing sample site location and appearance. Maps were created and adapted using ArcGIS 10.7.1.


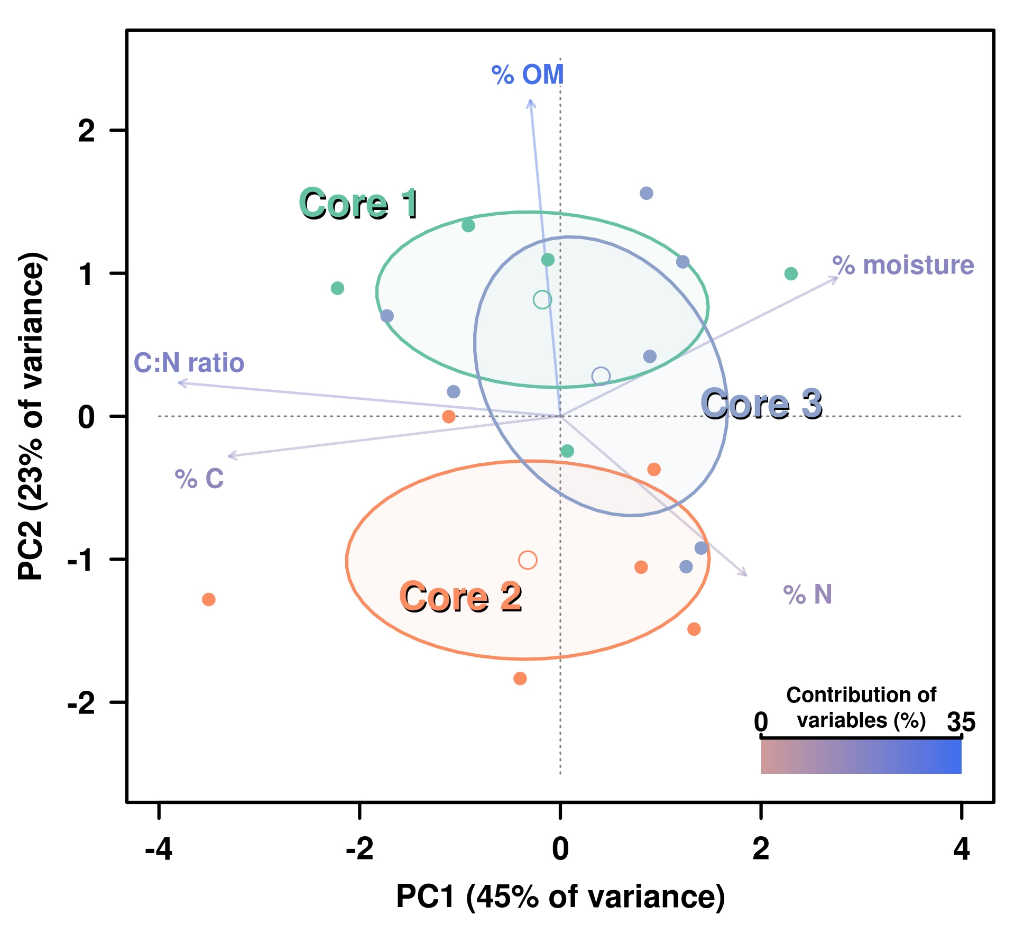


**Figure S2. PCA for Carbon-Nitrogen chemical properties.** Analysis by Principal Components Analysis (PCA) of the influence of chemical properties related to concentration of carbon and nitrogen in the sediment. Contribution of each variable (chemical properties) to this graphical representation is shown by a color key from light pink (less contribution) to blue (highest contribution). In this case, percentage of organic matter (% OM) shows the biggest contribution to the sample distribution. Ellipses and open dots represent variance and mean measured for each core, respectively.


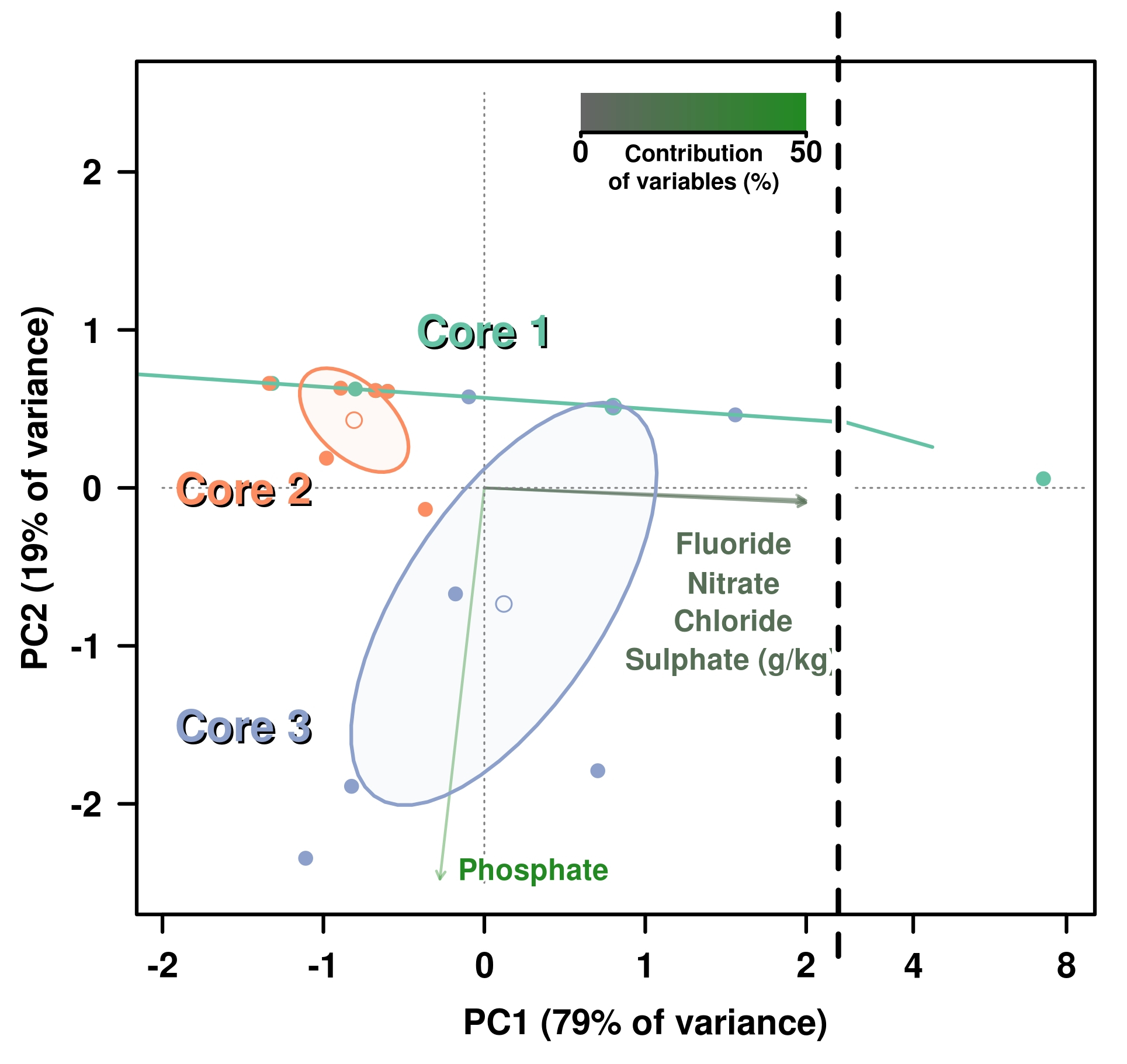


**Figure S3. PCA for anion concentration.** Analysis by Principal Components Analysis (PCA) of the influence of chemical properties related to the concentration of anions in the sediment. Contribution of each variable (chemical properties) to this graphical representation is shown by a color key from medium grey (less contribution) to light green (highest contribution). In this case, concentration of phosphate shows the biggest contribution to the samples distribution in this PCA. In addition, an overlapping can be observed for the other anions due to the proportions being similar between the three cores. Note that sample 1.3.2 (far right symbol for Core 1 shows much higher concentrations of fluoride, chloride, nitrate and sulfate, relative to the rest of the layers. Therefore, a dashed line is cutting the x-axis to mark the change of scale. Ellipses and open dots represent, variance and mean measured for each core, respectively.


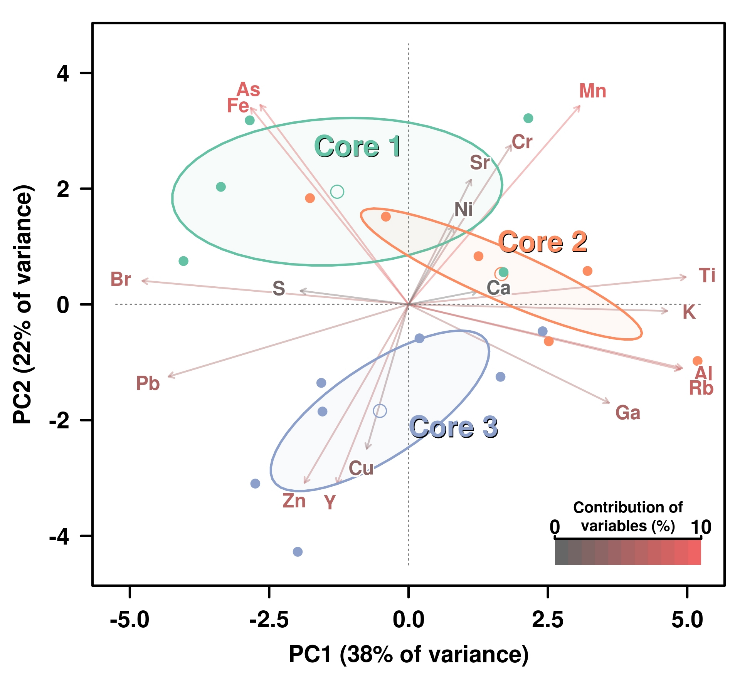


**Figure S4. PCA including elements concentrations as variables.** Analysis by Principal Components Analysis (PCA) of the influence of chemical properties related to concentration of elements and heavy metals. Contribution of each variable (chemical properties) to this graphical representation is shown by a color key from grey (less contribution) to light red (highest contribution). In this case, highest contribution is shared between As, Fe and Mn. Ellipses and open dots represent, variance and mean measured for each core respectively.

**Data Availability Statement**

The datasets generated for this study can be found in the National Center for Biotechnology Information (NCBI) with a Bioproject accession number PRJNA639793, the Biosample accession numbers SAMN15249365-15249398, and the Accession numbers SRP12024101-12024128. Details are available in the Supplementary Table S4.
